# Perspectives of Triage Team Members Participating in Statewide Triage Simulations for Scarce Resource Allocation During the COVID-19 Pandemic in Washington State

**DOI:** 10.1001/jamanetworkopen.2022.7639

**Published:** 2022-04-18

**Authors:** Catherine R. Butler, Laura B. Webster, Douglas S. Diekema, Megan M. Gray, Vicki L. Sakata, Mark R. Tonelli, Kelly C. Vranas

**Affiliations:** 1 Division of Nephrology, Department of Medicine, University of Washington, Seattle; 2 Veterans Affairs Health Services Research and Development Center of Innovation for Veteran-Centered and Value-Driven Care, Seattle, Washington; 3 Bioethics Program, Virginia Mason Medical Center, Seattle, Washington; 4 Department of Bioethics and Humanities, University of Washington School of Medicine, Seattle; 5 Department of Pediatrics, University of Washington School of Medicine, Seattle; 6 Trueman Katz Center for Pediatric Bioethics, Seattle Children’s Research Institute, Seattle, Washington; 7 Northwest Healthcare Response Network, Seattle, Washington; 8 Division of Pulmonary, Critical Care and Sleep Medicine, Department of Medicine, University of Washington, Seattle; 9 Center to Improve Veteran Involvement in Care, VA Portland Health Care System, Portland, Oregon; 10 Division of Pulmonary and Critical Care Medicine, Department of Medicine, Oregon Health and Science University, Portland

## Abstract

**Importance:**

The COVID-19 pandemic prompted health care institutions worldwide to develop plans for allocation of scarce resources in crisis capacity settings. These plans frequently rely on rapid deployment of institutional triage teams that would be responsible for prioritizing patients to receive scarce resources; however, little is known about how these teams function or how to support team members participating in this unique task.

**Objective:**

To identify themes illuminating triage team members’ perspectives and experiences pertaining to the triage process.

**Design, Setting, and Participants:**

This qualitative study was conducted using inductive thematic analysis of observations of Washington state triage team simulations and semistructured interviews with participants during the COVID-19 pandemic from December 2020 to February 2021. Participants included clinician and ethicist triage team members. Data were analyzed from December 2020 through November 2021.

**Main Outcomes and Measures:**

Emergent themes describing the triage process and experience of triage team members.

**Results:**

Among 41 triage team members (mean [SD] age, 50.3 [11.4] years; 21 [51.2%] women) who participated in 12 simulations and 21 follow-up interviews, there were 5 Asian individuals (12.2%) and 35 White individuals (85.4%); most participants worked in urban hospital settings (32 individuals [78.0%]). Three interrelated themes emerged from qualitative analysis: (1) understanding the broader approach to resource allocation: participants strove to understand operational and ethical foundations of the triage process, which was necessary to appreciate their team’s specific role; (2) contending with uncertainty: team members could find it difficult or feel irresponsible making consequential decisions based on limited clinical and contextual patient information, and they grappled with ethically ambiguous features of individual cases and of the triage process as a whole; and (3) transforming mindset: participants struggled to disentangle narrow determinations about patients’ likelihood of survival to discharge from implicit biases and other ethically relevant factors, such as quality of life. They cited the team’s open deliberative process, as well as practice and personal experience with triage as important in helping to reshape their usual cognitive approach to align with this unique task.

**Conclusions and Relevance:**

This study found that there were challenges in adapting clinical intuition and training to a distinctive role in the process of scarce resource allocation. These findings suggest that clinical experience, education in ethical and operational foundations of triage, and experiential training, such as triage simulations, may help prepare clinicians for this difficult role.

## Key Points

**Question:**

What are the experiences of individuals participating in scarce resource triage teams during the COVID-19 pandemic, and how can clinicians prepare for this role?

**Findings:**

In this qualitative study of 41 triage team members participating in multi-institutional triage simulations in Washington state, participants described how they grappled with clinical uncertainty and ethical challenges and how the triage task could conflict with professional values and required transformation of the usual clinical mindset.

**Meaning:**

These findings highlight challenges that triage team members may face and suggest that clinical experience, education in ethical and operational foundations of triage, and experiential training may help prepare them for this difficult role.

## Teaser

This qualitative study investigates themes in the experiences of triage team members participating in statewide simulations of health care triage under circumstances of scarce resource allocation during the COVID-19 pandemic in Washington State.

## Introduction

Surges in patient volumes during the COVID-19 pandemic have resulted in severe shortages in health care resources^1,2,3^ and declaration of crisis capacity in several states in the United States.^4,5^ Most planned protocols for allocating intensive care resources are intended to identify and prioritize patients with the greatest likelihood of survival to receive scarce resources.^6,7,8,9,10,11^ While algorithms and organ failure scores have been proposed as strategies to categorize patients by estimated prognosis as part of triage plans, there is increasing concern about the effectiveness and fairness of these automated selection mechanisms.^12,13,14,15,16^ Many allocation systems ultimately rely on clinician judgment to make final prognostic determinations.^13,17^

During the COVID-19 pandemic, clinicians have reported feeling ill prepared to take on the task of triage in crisis capacity settings.^18^ Few clinicians receive formal education or training in approaches to health care triage.^19^ In fact, clinicians are typically trained to explicitly exclude considerations of resource limitation in clinical decision-making.^20^ Nonetheless, preparation for crisis capacity has focused on the rapid deployment of triage teams, with little attention to strategies to prepare and support these team members.^21^ We aimed to elucidate the experiences and perspectives of clinicians participating in triage simulations during the COVID-19 pandemic in order to identify opportunities to better support these teams in real-world settings and to improve the allocation process.

## Methods

This qualitative study was determined to be exempt from full review and requirement for informed consent because it did not constitute human participants research under the Common Rule by the Benaroya Research Institute Virginia Mason Institutional Review Board. Participants were not financially compensated. We reviewed the Consolidated Criteria for Reporting Qualitative Research (COREQ) reporting guideline in reporting the details of our methodology.

We completed a 3-phase research program to develop and test a scarce resource triage team process in Washington state during the COVID-19 pandemic. First, we conducted a Delphi study of emergency preparedness experts to develop a list of patient information items needed to inform triage team decision-making.^22^ Second, we held multi-institutional triage team simulations to evaluate efficiency, consistency, and effectiveness of the approach.^23^ Third, we conducted a qualitative study of the triage team process and experiences of team members. In this report, we describe the results of this third component of the research program.

### Triage Simulations

As previously described,^22,23^ clinicians and ethicists involved in Washington state institutional emergency preparation participated in triage team simulations from December 2020 to February 2021. Triage teams consisted of at least 2 clinicians (physicians and nurses) and a team member with formal training in bioethics or experience in clinical ethics consultation.^12^ Health care institutions were encouraged to refer senior clinicians and ethicists who had volunteered to participate in institutional triage teams if this were to be needed during the pandemic.

Triage team participants engaged in a 60-minute orientation session on ethical and operational aspects of triage, a 90-minute triage simulation, and a 30-minute debriefing session. During the simulation, teams reviewed a limited set of deidentified patient information items (eFigure 1 in the Supplement) and assigned each patient case to 1 of 5 prognostic categories (eFigure 2 in the Supplement). Participants also completed a survey of their demographic information and clinical background. Self-identified race and ethnicity were ascertained because these features are likely associated with participants’ personal and professional experience and perspective. Participants were asked to report their race (American Indian or Alaska Native, Asian, Black or African American, Native Hawaiian or other Pacific Islander, White, prefer to self-describe, or prefer not to say) and whether or not they were Hispanic or Latino.

### Data Collection: Simulation Observation and Semistructured Interviews

Simulations were silently observed by at least 3 members of the study team who took notes on team dynamics and decision-making. We used a purposive sampling approach to recruit participants for follow-up interviews, which included sampling across different simulation sessions, clinical backgrounds, and triage team roles (ie, clinician vs ethicist).^24^ Interview participants completed a one-on-one 30-minute to 60-minute audio-recorded interview with C.R.B. (a nephrologist and researcher with expertise in qualitative methodology) or L.B.W. (a bioethicist and emergency medicine [EM] and critical care registered nurse). A semistructured interview guide was developed by C.R.B. and L.B.W. and included open-ended questions to elicit participants’ perspectives and experiences pertaining to triage team simulations (eTable in the Supplement). Throughout the period of data collection, approach to recruitment and the interview guide were iteratively refined by C.R.B. to support thematic saturation.^24,25^

### Data Analysis

We conducted an inductive thematic analysis^26^ of observation notes and written interview transcripts to identify emergent themes describing participants’ perspectives and experiences on triage simulations. Two team members with expertise in qualitative analysis (C.R.B. and K.C.V., the latter being an intensive care physician and researcher) independently reviewed and openly coded observation notes and transcripts until reaching thematic saturation (the point at which no new concepts were identified).^24,27^ Both team members continued to review and code remaining transcripts to identify additional exemplar quotations. Throughout analysis, the 2 investigators used a constant comparison^19^ approach to iteratively review codes, collapsed codes into groups with related meanings and associations, and developed larger thematic categories.^24,26^

All coauthors (C.R.B.; K.C.V.; L.B.W.; D.S.D., a bioethicist and pediatric EM physician; V.L.S., a leader in Washington disaster preparedness and an EM physician; and M.R.T., a bioethicist and critical care physician) reviewed exemplar quotations and themes and together developed the final thematic schema. We used Atlas.ti software version 8 (Scientific Software Development) to organize and store text and codes.

## Results

Among 41 clinicians and ethicists (mean [SD] age, 50.3 [11.4] years; 21 [51.2%)] women) who participated in 12 triage simulations, there were 5 Asian individuals (12.2%), 35 White individuals (85.4%), 1 individual with more than 1 race or ethnicity, and no individuals with other race responses, which included American Indian or Alaska Native, Black or African American, Native Hawaiian or other Pacific Islander, preferred to self-identify, or preferred not to say; there was 1 Hispanic or Latino individual (2.4%). Most participants were working in urban hospital settings (32 individuals [78.0%]), and the group had a mean (SD) 20.9 (11.5) years of health care experience (Table 1). In addition to data collected from simulation observation notes, we completed follow-up interviews with 21 participants to reach thematic saturation.

**Table 1.** Participant Characteristics

Characteristic	Participants, No. (%)	
	Triage team simulation (n = 41)	Interview (n = 21)^a^
Age, mean (SD), y	50.3 (11.4)	52.0 (13.2)
Race		
Asian	5 (12.2)	4 (19.0)
White	35 (85.4)	16 (76.2)
More than 1 race	1 (2.4)	1 (4.8)
Other responses^b^	0	0
Hispanic or Latino ethnicity	1 (2.4)	1 (4.8)
Women	21 (51.2)	13 (61.9)
Men	20 (48.8)	8 (38.1)
Years in clinical practice, mean (SD), y	20.9 (11.5)	22.7 (13.5)
Type of primary institution		
Academic	10 (24.4)	5 (23.8)
Private	7 (17.1)	4 (19.0)
Community	22 (53.7)	11 (52.4)
Other	2 (4.9)	1 (4.8)
Primary practice setting		
Urban	32 (78.0)	18 (85.7)
Rural	6 (14.6)	2 (9.5)
Other	3 (7.3)	1 (4.8)
Primary work site^c^		
Clinic or outpatient	6 (14.6)	4 (19.0)
Acute care	20 (28.8)	12 (57.1)
Intensive care	9 (22.0)	4 (19.0)
Emergency department	6 (14.6)	4 (19.0)
Nonclinical setting	5 (12.2)	3 (14.3)
Other setting	4 (10.0)	0
Clinical ethics experience	19 (46.3)	11 (52.4)

^a^ Interview participants are a subset of all triage team simulation participants.

^b^ Other race responses were combined in the table given that no individuals chose these responses. These options included American Indian or Alaska Native, Black or African American, Native Hawaiian or other Pacific Islander, prefer to self-identify, and prefer not to say.

^c^ Responses were nonexclusive.

Three interrelated themes emerged from qualitative analysis: (1) understanding the broader approach to resource allocation, (2) contending with uncertainty, and (3) transforming mindset (Figure). Exemplar participant quotations for themes and subthemes are included in Table 2.

**Figure.**
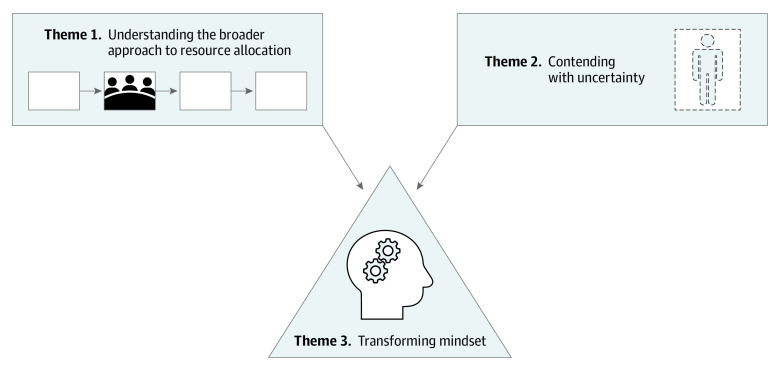
Thematic Schema Illustrating Experiences in Triage Team Simulations Although the triage team’s task was defined narrowly around assigning patient cases to prognostic categories, team members sought to understand ethical and operational underpinnings of the entire triage process and grappled with clinical and ethical uncertainty. The team’s task drew on participants’ existing skills and experience but could also feel unfamiliar and even antithetical to their professional values and required a transformation of their usual approach to decision-making.

**Table 2.** Exemplar Quotations

Themes and subthemes	Participant profession	Exemplar quote
**Theme 1: understanding the broader approach to resource allocation**		
Upstream and downstream processes	Ethicist	“The part that’s the wildcard is the logistics of it. So what happens? Do I get paged? Are we doing this by phone? … How does this work within our system?”
	Emergency physician	“Who from our hospital is on that [triage] team? … Tough when we have one ethicist in [our region] and then a bunch of other clinicians with varying degrees of interest or abilities in ethics training who are all working full time.”
	Emergency physician	“When are resources being removed? … The attending physician [should] be protected from that because their duty is to the patient primarily. So the triage team might be the one that has to go tell the family, right?”
Ethical foundations and buy-in	Critical care physician	“There probably is some hierarchy of survival within the orange and yellow [prognostic category] group. That’s where, if this group gets randomized … that would be distressing. … In terms of trying to do the most good, I feel like, oh, maybe there should be some kind of secondary allocation.”
	Critical care and outpatient physician	“First, I think we had to kind of accept the validity of that [patient information] form. … The first couple we were kind of struggling with, ‘I sure wish I knew this. Can we ask for that? And what about this?’ But [we] very quickly fell into line.”
	Critical care physician	“[The triage process] is definitely imperfect. …There are many things that we work with in medicine that are imperfect and they’re the best we have. And if we were in a true triage situation, I personally think we would all be best served to work under the same ground rules.”
	Emergency physician	“Seventy to 100 physicians are giving input into this [patient information form] and they’re all from different specialties, and fairly rigorous discussion occurred around it. And so that gives me a little more confidence that the end result is well represented … knowing the process, knowing how it will all work, and that it’s fair for everybody.”
Locus of moral responsibility	Ethicist	“This almost feels like it’s battlefield triage. If you reach that point … it’s haunting. [If] I had the resource, I could’ve offered it, the person could’ve lived longer, but I have to make a choice. And those kind of choices come with a very heavy, heavy moral price.”
	Outpatient physician	“When you’re at the bedside … it’s very hard to make those decisions and it’s useful to have this team where … you’re basically impersonalizing the details.”
	Critical care physician	“It wasn’t deciding whether or not this person was going to get a ventilator or the last ICU bed, but more so what are this person’s chances of surviving until hospital discharge, which is something we do all the time. … The resource allocation happens in the next step. … Now that I’ve done the simulation, it would be easier for me to participate in the actual triage process.”
	Critical care physician	“I think it was good that we didn’t know how many resources were available. … It would be hard not to keep in the back of your head, like, ‘Gosh, how many people did we put in red already? And how many do we have left? … What if there’s a really young person down the line who really could benefit?’”
	Critical care physician	“Human nature, I guess. I think when you’re making these decisions you can’t divorce yourself from the implications. … I kind of wrestled with that after the sim[ulation]. Would I want to know more about what the on-the-ground situation was? Like how many ICU beds and ventilators? … I’m not sure.”
	Emergency physician	[Regarding randomization as a tiebreaker within prognostic categories] “At the very moment when you need to make the decision of who gets it and who doesn’t, to randomize it feels like you’re kind of letting go of the principles that you founded it on. …We need humans to make this decision because it’s that important.”
	Acute care physician	“If you do get to that point … how do we tell the public that we’re doing this? … I think if it’s coming from a state level to start with, it gives it some more authority.”
**Theme 2: contending with uncertainty**		
Lack of clinical detail	Critical care physician	“More information can lead to decision paralysis. … As much as I want more information, there are many aspects that would be difficult to ascertain accurately, and then you introduce a whole other level of bias that may lead to incorrect or inappropriate decisions.”
	Acute care physician	“It’s just data points right, so you just have to kind of open your mind. … Try to put together the pieces and I think that’s why it’s important to have clinicians who, I think, are actively involved in clinical care because when you see these patterns you get an idea of like, ‘Oh, this is probably the patient that’s at this point’ … a context for how that story would play out.”
	Ethicist	“The very first one we did, we were immediately assuming that the pronouns were he/him, and we didn’t know that person’s gender. … When you make up a picture of a patient in your mind and you’re assigning a specific sex or gender to them, there are going to be assumptions that go along with that.”
	Critical care and outpatient physician	“I felt pretty blind … just the lack of information … [the patient] sat right in the middle of this gray area, I have no idea how this guy’s going to go. I have no idea why he’s here in the hospital. … I don’t know what his prognosis is.”
	Acute care and outpatient physician	“I couldn’t figure out what exactly was happening with the patient, and therefore I couldn’t triage their survivability. It’s like when the ER doc calls you with a consult and they give you a two-sentence [report] … and then, sometimes they tell you miscellaneous information. … The times I felt like I couldn’t make a decision, most of the time it was because a story didn’t make sense.”
	Critical care physician	“It’s disembodied, right? … You look through the window and … eyeball somebody. They look good or they don’t look good. … Hard to say what the actual thing is that’s triggering some prognosis estimate. … I’ve done that for so many years that when you’re removed from it, all of a sudden, you miss it. … It’s all the intangibles. … That’s what we do. … Some of it is completely incalculable.”
	Critical care and outpatient physician	“I don’t know who filled out the little [patient information] sheet. That sounds silly, but you need someone with enough acumen to review the medical record. … People would need assurance that was happening accurately.”
	Emergency physician	“As I’ve done the [triage team simulations] … I put value in what the bedside clinicians are telling me they’re seeing. And I know that’s biased and it’s based on training and experience. … But I still value it because they know the patient. … It balances out the numbers on the page.”
	Emergency physician	“It felt like we were using more anecdotal, like, ‘Oh I’ve seen this person survive to hospital discharge,’ … ‘I know a good 80-year-old,’ or ‘My mom’s 80 years old.’ … That seemed to kind of melt away a little bit toward the end … and it was more like, ‘65-year-old, diabetic. They’re a yellow.’ Kind of clearer, but also colder.”
Ethical ambiguity	Critical care physician	“The ethicist [triage team member] was a little, almost superfluous in the real moment. I think in the planning of the tool … and the assuring that it’s deidentified and doesn’t have any hint of having any emphasis on socioeconomic status or whatever … the ethicist’s input is critical … [but] it would be the uncommon case in these real-life cases where, from the data that you’re given, there’d be any ethical consideration to discuss much.”
	Acute care and outpatient physician	“I could not even have imagined trying to sit down … [and say], ‘How do we decide on who gets what?’ It was nice to have something handed off to you. … People much smarter than I am have already been thinking about this and creating a framework for this, and now really our duty is to really enact that in our system.”
	Critical care physician	“I think we can all agree that two lives are more valuable than one. … A pregnant person with COVID? So we know their risk of death is higher … [but] I would tend to say that if that person was on the line between a higher category or a lower category, I would go higher.”
	Acute care nurse	“[The ethics team member] was saying the quality of life is that person’s experience in it. But then, if you look at somebody who might survive neurologically … vs somebody who survived but would be neurologically extremely impaired. … How do we think about that? … Maybe it’s really the framework of utilitarian[ism], where you would say … ‘Yes, they will survive discharge, but they’re going to be in the hospital a year.’ … It’s a resource utilization [issue]. … If you’re really in dire straits, maybe that’s a factor.”
Imperfection in a consequential task	Ethicist and nurse	“We’re making really important decisions that are going to have life and death consequences. … As good clinicians, it is our obligation to make sure we’ve gathered all of the relevant information. [But] somebody else has already decided for us what the relevant information is, and that’s all we get. And that feels like a violation of some of our basic obligations as clinicians.”
	Critical care physician	“When there was uncertainty, I think our bias was to just go toward the higher group. … I’d rather overestimate someone’s chance of survival than underestimate.”
	Critical care physician	“Getting comfortable with uncertainty … I can imagine that that would freeze a lot of people. I think the it’s sort of the difference between an outpatient internist vs an EM doctor. So it’s that ability to incorporate imperfect information and make a decision even though you know that you might not be right.”
	Critical care physician	“We have no way of knowing if our predictions are true. … You want to be perfect in the moment … [because] it’s so important to get it close to right.”
	Acute care and outpatient physician	“[A prior simulation] was distressing for me. … [But the instructor said,] ‘We’re going to make it better.’ So my sense is, stay tuned and we’ll have more simulations, more practice. Practice to make better, not perfect.”
**Theme 3: transforming mindset**		
Disentangling bias from clinical thinking	Critical care physician	“They tried to teach us about unconscious bias and removing ourselves from it, and I have mixed feelings about it. I think there are definitely some areas where we shouldn’t be letting biases enter into our decision-making. There’s other things that kind of bleed into overall assessments that I think maybe we ought to be continuing to incorporate.”
	Ethicist	“It was unclear as to whether age was supposed to be a factor. And what I assumed. … If it wasn’t a significant factor that impacts triage and prognosis, it would not be in here. … I was trying probably too much to be conscious not to demonstrate any kind of age bias.”
	Critical care physician	“One of the stroke patients, maybe had a pretty bad stroke, but so what? Who were we to judge? We kind of verbalized, ‘Gosh, I know what I wish I could say about this because this person has a pretty grim future from my standpoint, [and] I wouldn’t want to live like that. But let’s put that aside. This person can live.’”
	Critical care and outpatient physician	“[The ethicist] did a nice job of calling out the risk of agism. … She’d sort of say, ‘Does it matter?’ And I think we’d squirm a little, and sometimes it sort of did matter … [but] 62 and 67? Not so much.”
	Critical care and outpatient physician	“It’s good to have a group [triage team]. … We were in a deliberative process and were able to discuss what that might be and what the best answer was going forward. … I think you’re more at risk that 1 or 2 [triage clinicians] can spin a story and convince themselves of it.”
	Ethicist	“I’m not a physician, [and] … I don’t share physician blind spots … things that might just go unspoken because you all assume the same things. … So I can stop and ask a question, just in case any of the unspoken things are things that deserve more consideration.”
A task antithetical to usual practice	Critical care physician	“We tend to do … a lot more in terms of … quality of life at discharge and beyond. … What post-ICU life is going to look like if they survive and whether or not that would be in line with the patient’s goals of care. But it’s kind of hard to kind of take myself out of that role.”
	Acute care and outpatient physician	“Both [physicians] agreed that this person was probably not going to return back cognitively, but the question we’re being asked is will they survive to discharge? I remember I said I don’t have enough information, I’d really have to talk to neuro[logy].”
	Ethicist	“Some kind of robot or computer algorithm [can’t] decide this. … If you could somehow take the human judgements and values and unspoken things out of it … that’s not medicine. As much as we’d like to think it’s all based on numbers, it’s still a very human enterprise. …We’re the ones who make the numbers mean something.”
	Acute care and outpatient physician	“If someone is going to go without and someone is on a ventilator, is my job to make the recommendation this person comes off the ventilator. … I’m OK with this person not getting a ventilator, but they do need palliative care … and if I don’t know that they’re going to get it, I don’t feel good about it. I think my job is, our task as the … [triage team] has been incomplete. We’re just passing out ventilators and feeling horrible about it.”
	Emergency physician	“One of the ways that I’m going to be able to sleep at night is by knowing that I wrestled with it. … It helps me feel like we’re really honoring the patient in a way that can’t be honored when you just adhere to an algorithm. … That struggle and that investment makes the process more humane and more palatable.”
	Emergency physician	“We could just have a computer do this work, but we have chosen specifically not to. And I think the reason is because of that human connection. So if you start to act more and more like algorithmic, or think more in terms of computer because you’re tired or because you just want to get it done, that’s not the point.”
Importance of open deliberation	Emergency physician	“I would trust that … you have the patient’s best interest at heart. And you’re looking at this through the lens of your training, … your objectiveness, as well as your subjectiveness and your empathy.”
	Acute care physician	“Not knowing [other triage team members’] … scope of practice is probably the first major limitation because you just don’t know, well, what kind of patients do you take care of? Can I trust you to accurately triage a surgical patient? … [But] once we started talking, I could understand that she saw patients probably in the ICU and she saw postop[erative] patients, so I had some understanding of what her capacity was based on … the questions she asked, the scenarios that came up, and her comments.”
	Acute care physician	“I felt a little intimidated ‘cause I am early in my career, and I’m … not as experienced as a lot of the other members. … So, I was nervous [about] if I would do a good job or not. But because it was a nice conversation and open question-answer, I felt like I could still speak up and voice my concerns.”
	Emergency physician	“It should not be an individual decision. It’s just too weighty. But when you hear everyone else talk it out, there’s a comfort in that. There’s a comfort when the team gets to relative consensus. There’s a relief.”
	Ethicist and nurse	“[As the ethicist team member,] if I get engaged in having an opinion about where this person should be triaged, then I lose my ability to look at it from the outside and ask good questions. … A lot of the work we do in [ethics] consults … bump[s] right up against our clinical roles. But again, to really be clear about [it], that’s somebody else’s role in this moment, that’s not my role.”
	Ethicist and nurse	“I don’t think that’s my [ethicist] role, to make decisions. It’s to sort of poke people and get them to … come to a resolution. … My role is to notice when they’re struggling. My role is to see if there are questions I could ask that would help clarify their thinking.”
	Acute care physician	“[The ethicist team member] kind of had more like a bird’s-eye view ‘cause she wasn’t making decisions each time. … She pointed out what we had previously … triaged people as and how it might have related to the one we were actively discussing. … [She] helped us … think about the underlying thought process with her comments and questions.”
	Ethicist and nurse	“The first cases where I began to feel like, OK, there’s room for me [as an ethicist] were the ones where they got really stuck. … My ability to then say, ‘So, it sounds like you disagree about this.’ Or, ‘It sounds like one of you is saying this, but the other one doesn’t think you have enough information.’ … It’s mediation work.”
	Critical care physician	“[The ethics team member] was able to inject a structure to some of the thinking processes … saying, … ‘Is that consistent with what we’re trying to do?’ … Her questions really prompted us to think about it more clearly.”
Need for experience and practice with triage	Critical care physician	“This is not something you’re inherently able to do. … There is probably a learning curve, so thinking about this prospectively is important. Being thrown into that situation, we certainly make do, but I think it would be better to have a little bit of experience.”
	Outpatient physician	“I trained in India and we’ve triaged. … To me, it’s not hard to be in a triage position. … I know, unfortunately, you can’t save the world, right? So for me, it’s kind of easier in my mind to say, ‘OK, I have to shift to this.’ I think it’s easier than for somebody who’s never done it.”
	Critical care physician	“Having to decide whether someone … gets an ICU bed, doesn’t get an ICU bed … we do kind of do that already. … If there’s a patient who I feel like, gosh, a goals of care discussion is warranted here before we go down this pathway, in this 95-year-old person with advanced dementia who’s coming in with respiratory failure.”
	Emergency physician	“The simulations are the best thing. … I remember being a resident. … I had to do a mock sim[ulation] of a pediatric code, and everything went to crap. … After that, I was like, well, I’m never going to be caught with my pants down again, so I would run simulations in my head so I prepared my brain for what I was going to have to do. … It’s not so anxiety-provoking.”

Abbreviation: EM, emergency medicine; ER, emergency room; ICU, intensive care unit.

### Understanding the Broader Approach to Resource Allocation

Participants in triage simulations strove to understand operational and ethical foundations of the triage process as a whole. They also strove to understand their roles and responsibility within this broader framework.

#### Upstream and Downstream Processes

Participants could be uncertain or concerned about whether and how the triage process would be operationalized in clinical settings. They identified practical barriers to enlisting triage team members, such as limited staff and clinician availability, and questioned how triage and resource reallocation decisions would be communicated to clinical teams and implemented. They also anticipated that triage decisions and responsibility to implement these decisions may contribute to moral distress among clinical staff and highlighted the relevance of trust and buy-in among these clinicians, as well as strategies to support well-being.

#### Ethical Foundations and Buy-in

Participants drew on their own experience and training in emergency response and bioethics to critically examine and question the design and ethical underpinnings of the triage process used in the simulation. Transparency about the triage process as a whole, including the process of development, operational features, and ethical justification, were seen as critical for fostering trust and cooperation from clinicians, triage team members, and the public. While participants may have disagreed with specific aspects of the triage approach, many participants also emphasized that they would and should accept the rules as defined to support consistency and fairness.

#### Locus of Moral Responsibility

Participants agreed with the value of having a separate triage process to protect bedside clinicians, but they also anticipated that serving as a triage team member would be emotionally taxing and distressing. Many participants found it difficult to avoid feeling personally responsible for the tragic implications of the work. Several physician participants even described a professional obligation to accept ultimate responsibility for their decisions. Explicitly acknowledging that the triage team was only one component of a larger process of scarce resource allocation helped alleviate some of this moral burden. Clear institutional and state support was also seen as necessary to legitimize the triage team’s work.

### Contending With Uncertainty

Triage team members grappled with how to make decisions with limited clinical and contextual information about patient cases. They also struggled with ethically ambiguous features of these patient cases.

#### Lack of Clinical Detail

While triage team members recognized the need to limit access to extraneous patient information that may have distracted from their task or evoked biases, some found it challenging to determine prognostic estimates with the limited clinical and contextual data available to them. Critical care experience, specifically caring for patients with COVID-19, was seen as necessary for supporting pattern recognition. Nonetheless, participants sometimes struggled to develop a cohesive narrative or story for each patient or decipher one scenario among a range of possible clinical scenarios. Clinicians cited the value of quick visual assessments, or “eyeballing,” patients in their usual practice to glean intangible prognostic information. Absent a discussion with a patient’s clinical team, clinicians were sometimes uncertain how to interpret or how much to trust subjective data available to them (eg, a report of the patient’s clinical trajectory).

Many participants described a shift in the pace and approach to team deliberation over the course of the triage simulation as participants became more familiar with the process. Later in the simulation, participants were able to make prognostic determinations more rapidly and patients were discussed in the abstract, with less effort to fill in missing contextual details.

#### Ethical Ambiguity

Multiple participants suggested that any ethical questions had been largely adjudicated during development of the triage process, leaving the triage team task itself relatively straightforward and dependent solely on clinical knowledge. However, others described deliberating over a range of ethical questions and uncertainties in the triage team simulation. While participants understood the triage team to be responsible for an intentionally narrow task of assigning prognostic categories, they grappled with other factors that were also felt to be ethically relevant to triage (eg, pregnancy status and duration of need for a limited resource). These features were sometimes used to nudge a patient case up or down in prognostic categories.

Clinicians found it helpful to refer back to ethical principles guiding triage to support critical reasoning through challenging patient cases. They appreciated input from ethicist team members to help clarify thinking and expose value conflicts.

#### Imperfection in a Consequential Task

Making important decisions with limited clinical information could feel irresponsible or neglectful of professional responsibilities to clinicians. Multiple participants described a default to select a more optimistic prognostic category when there was any residual prognostic uncertainty. Senior clinicians suggested that confidence resulting from years of clinical experience was valuable in making difficult decisions without becoming mired in details or indecision. Several identified the importance of simply acknowledging the unfamiliar and distressing nature of high-stakes decision-making with imperfect information to alleviate moral distress. While the triage process may not have been perfect, it offered a starting point for future iterative improvement.

### Transforming Mindset

Participants saw their clinical experience as valuable in making prognostic determinations. However, they also identified pitfalls of relying on their intuition and a need to substantially reshape their usual cognitive approach to patient care to better align with the unique task at hand.

#### Disentangling Bias From Clinical Thinking

Participants had been instructed to monitor for implicit biases, but they sometimes found it difficult or even contradictory to try to disentangle these biases from clinical judgment. They could be unsure of how to use information about patient characteristics, such as age and frailty, which were perceived to be associated with implicit biases, but also integral to prognostication.

Participants described active self-monitoring and explicit team discussions about how biases may shape decisions. The ethicist team member was especially active in monitoring for consistency and the association of implicit biases with outcomes of decision-making. Participants found the diversity of clinician and nonclinician team members’ perspectives to be helpful in guarding against individual biases and cultural assumptions within the medical profession.

#### A Task Antithetical to Usual Practice

Participants commented on how, in clinical practice, prognosis often constituted a more global assessment than physiologic survival, and this discrete outcome could be difficult to disentangle from considerations of quality of life. When considering patients with severe neurologic injury, some team members were confident in prognosticating about quality of life but felt unqualified to estimate likelihood of survival.

Understanding and making judgments grounded in the entire patient context, including patient goals and treatment preferences, was also seen as intrinsic to good medical practice. Participants sometimes caught themselves assuming an advocate role by arguing for the best conceivable prognosis for each patient case. Team members worked to segregate the narrowly defined triage team task from other clinical considerations through explicit self-reminders, team discussion, and monitoring by the ethicist team member.

While clinician team members recognized the need to adapt their usual cognitive approach, they also saw their work as an important opportunity to infuse humanity and compassion into the triage process and demonstrate respect for patients despite terrible circumstances. Some participants expressed concern about becoming too algorithmic or robotic as the simulation progressed.

#### Importance of Open Deliberation

When participants did not have preexisting relationships, they built trust in their teammates’ clinical skills and professionalism by observing them reason through cases. Participants recognized a potential tendency for more junior clinicians and ethicist team members to defer to senior clinicians and aimed to maintain an environment in which team members felt comfortable voicing opinions. Developing group consensus was also seen as a way of diffusing the moral burden of responsibility for a consequential task.

Clinician and ethicist team members described a role for the ethicist as a process facilitator and mediator. This team member often prompted clinicians to explain their reasoning aloud, which could motivate additional deliberation.

#### Need for Experience and Practice With Triage

Some team members felt comfortable with triage after working in under-resourced countries where health care rationing was routine. Other participants identified familiar aspects of their clinical jobs that required an analogous mindset, such as prioritizing patients to receive immediate care in a busy emergency department. However, many participants also commented on how work as a member of the triage team was intrinsically different from their usual clinical role, and several team members suggested that participation in the simulation study itself had served as important practice and preparation for a real-world triage scenario.

## Discussion

Thematic analysis of triage team simulation observations and interviews with participants in this qualitative analysis revealed a clinically, operationally, and ethically complex process of triage team deliberation. During the COVID-19 pandemic, clinicians across a range of health care settings have struggled to adapt clinical decision-making to the realities of resource limitation.^1,2,13^ While triage team participants were presented with a seemingly narrowly defined task focused on fair allocation of a scarce health care resource across the population, they nonetheless were reluctant to shift their focus entirely from patient-centered considerations and endeavored to maintain compassion for individual patients in the triage process. The teams’ task drew on participants’ existing skills and experience, but the task could also feel unfamiliar and even antithetical to participants’ professional values and required a transformation of their usual approach to decision-making.

Triage team members’ clinical experience, especially in critical care, was seen as important to support pattern recognition with limited information, comfort making decisions amid uncertainty, and acceptance of a morally weighty task. However, in addition to factual clinical knowledge, this work emphasized the relevance of how team members think and ways in which the deliberative process itself may support an effective team.^28,29,30^ Patient cases presented to triage team participants were stripped of the usual rich contextual detail that informs unconscious pattern recognition and clinical intuition.^31,32^ For this reason and because team members were explicitly asked to monitor for their own biases, participants were required to engage in a more analytical and self-aware approach to decision-making.^31^ Patient cases involving clinical and ethical uncertainty required critical thinking and reasoning.

Participation on hospital triage teams is likely to evoke ethical conflict and moral distress, not only because of the tragic circumstances under which these teams are deployed, but also because clinicians are asked to perform a task that may deeply conflict with their training and professional values (Table 3).^33,34,35^ This tension is reflected in the broader experiences of clinicians working during the pandemic.^36^ Moral distress has been associated with significant burnout and degraded mental health among clinicians during and before the COVID-19 pandemic and likely poses a challenge to preserving both triage team membership and the broader work force.^37,38^

**Table 3.** Sources of Ethical Conflict or Concerns and Potential Alleviating Factors^a^

Theme	Sources of ethical conflict or concern	Alleviating factors for ethical conflict
1. Understanding the broader approach to resource allocation	Moral responsibility for consequences of triage Tragedy of being unable to provide for all patients who deserve care	Explicit identification and acknowledgment of the tragedy of resource scarcity, conflicting duties to patients and populations, and appropriateness of moral distress Recognition that the triage team is only one part of a larger process that is designed to support fair allocation Diffusion of personal responsibility via team-based approach
2. Contending with uncertainty	Imperfection and uncertainty when making life and death decisions may feel irresponsible	Clinical experience, especially critical care and work with patients with COVID-19, may be associated with improved patient case pattern recognition and ability to make decisions despite limited information and residual uncertainty Team-based decision-making and incorporation of multiple clinician and nonclinician team member perspectives may be associated with lessened impact of individual biases and cultural assumptions
3. Transforming mindset	Clinical intuition, training, and professional values may conflict with triage team task	Education and simulation in triage processes and bias reduction training may be associated with a more analytic (vs intuitive) cognitive approach to decision-making Team members with expertise in bioethics may help navigate value conflicts and monitor the deliberative process for consistency

^a^ Sources of ethical concerns for triage team members, as well as potential alleviating factors, cut across themes that emerged from qualitative content analysis.

Insights from this work suggest opportunities to support triage team members and to improve the deliberative process. First, equipping team members with a broad understanding of clinical, operational, and ethical foundations of the entire process of resource allocation^17,39^ may help them to understand their specific role and may help alleviate the burden of personal moral responsibility. Second, an ethicist or team facilitator may serve a valuable role in mediating and monitoring deliberation to ensure that the team operates under consistent principles and goals.^40^ Diversity of perspectives and a trusting team environment may also be associated with improved active discussion and carefully considered decisions. Finally, our findings suggest that advance training should include cognitive approaches to bias reduction^41^ and basic bioethical reasoning. While it may be difficult to fully prepare clinicians for the reality of participating in triage,^18^ practice in simulations may help team members apply and develop skills. Simulated practice may also support clinicians in being more willing to volunteer^42,43^ and able to quickly adapt to an unfamiliar role.^21^

### Limitations

Our study has several limitations. First, this high-fidelity simulation was conducted among clinicians embedded in an ongoing health care emergency, but the experience of participating in a true triage scenario may differ. The roles and composition of triage teams may also differ among scarce resource allocation plans,^12^ and the process should be adapted to unique institutional and regional contexts. Mirroring the racial composition of physicians in Washington state,^44^ the majority of participants in our study were White, and findings may not reflect the perspectives of clinicians with different racial backgrounds.

## Conclusions

Thematic analysis of observation and interviews with participants in multi-institutional triage team simulations suggests that there is value but also challenges to the triage team model as a component of scarce health care resource allocation. These findings highlight the need for triage team members to have extensive clinical experience and other expertise, such as bioethics training. Education in ethical and operational foundations of scarce resource allocation and experiential training, such as triage simulations, may help prepare team members to perform a challenging task in advance of clinical deployment.

## Notes

Supplement. **eTable.** Sample Semistructured Interview Guide **eFigure 1.** Example Patient Information Form as Viewed by the Triage Team **eFigure 2.** Prognostic Category Descriptions as Viewed by the Triage Team
