# Supplementary material for: Perspectives of Triage Team Members Participating in Statewide Triage Simulations for Scarce Resource Allocation During the COVID-19 Pandemic in Washington State
Source: JAMA Netw Open. 2022 Apr 18;5(4):e227639. doi: 10.1001/jamanetworkopen.2022.7639 (PMC9016492; doi:10.1001/jamanetworkopen.2022.7639)
Supplement: Supplement. — eTable. Sample Semistructured Interview Guide eFigure 1. Example Patient Information Form as Viewed by the Triage Team eFigure 2. Prognostic Category Descriptions as Viewed by the Triage Team [file jamanetwopen-e227639-s001.pdf]

Supplemental Online Content

Butler CR, Webster LB, Diekema DS, et al. Perspectives of triage team members participating in statewide triage simulations for scarce resource allocation during the COVID-19 pandemic in Washington state. *JAMA Netw Open.* 2022;5(4):e227639. doi:10.1001/jamanetworkopen.2022.7639

eTable. Sample Semistructured Interview Guide

eFigure 1. Example Patient Information Form as Viewed by the Triage Team

eFigure 2. Prognostic Category Descriptions as Viewed by the Triage Team

This supplemental material has been provided by the authors to give readers additional information about their work.

© 2022 Butler CR et al. *JAMA Network Open.*

eTable. Sample Semistructured Interview Guide

Introduction

Before we start, I'd like to review that this study is covered by the Virginia Mason QUIP and reviewed by their IRB. Anything you say will be kept confidential among the study team.

How much time do you have to spend with me? It would be helpful for me to record so that I can concentrate on what you're saying rather than taking notes. Is it ok if I record? Do you have any questions before we start?

Semi-structured interview questions:

Generic probes:

- Can you tell me more about that?
- What do you mean by that?
- Can you give me an example of that?

1. So that I understand your background, can you tell me a little bit about your usual clinical, leadership, or other hospital roles?

- (If experience with institutional planning) What has it been like to plan for resource limitation at your institution?

2. Tell me about what it was like to work on the triage simulations.

- What was that like?
- What was challenging? What went well?
- What felt familiar? What felt different?
- What was surprising or unexpected?

3. How did the triage process go?

- How did you come to a decision?
- How was this similar or different than other clinical decision-making?

4. How did the group work together?

- What was challenging? What went well?
- What roles did different team members play?
- What was your role and how did you do this? What helped you and/or what acted as a barrier to your role?
- What makes a good team member?

5. How do you think this triage approach would go in the real world at your institution?

- How did this feel different than it might in real world?
- How do you see this process being integrated into practice?

© 2022 Butler CR et al. *JAMA Network Open.*

6. What about the triage process was familiar or different compared with your usual clinical practice?

7. Knowing what you do now, is there anything you would do differently?

8. What else haven't we covered or what haven't I brought up?

© 2022 Butler CR et al. *JAMA Network Open.*

eFigure 1. Example Patient Information Form as Viewed by the Triage Team

Please review the available information for this patient

**Triage Tracking ID:**	417Za3958D756 and *record #: RED - COVID-SAMPLE*
**Patients Age:**	35 ______
**Patient's Preferences:**	Wants ALL medically appropriate ICU treatments OR UNKNOWN ____

PAST MEDICAL HISTORY: NO known SEVERE or END-STAGE conditions in medical history

Blank lines = No data entered			
**Chronic Lung Disease**	______	**Chronic Kidney disease**	______
**Heart Failure**	______	**Chronic Liver Disease**	______
**Coronary Artery Disease**	______	**Malignancy**	______
**Other SEVERE or END-STAGE:**	______		
**Striped Category:**	______		

Check to ensure free text information entered is objective, clinically relevant to survival to discharge, and based on the best evidence and clinician judgment.

**Is death within 6 months is expected** *(from either an underlying terminal/end-stage condition or irreversible cause rendering death imminent)* **based on the best evidence and clinician judgment?**	**NO. This patient is expected to live LONGER than 6 months.**

Patient's Clinical Status:

**Days since patient was first hospitalized:** *(Transfers use 1st date)*	12
**Primary reason for hospital admission:**	COVID-19+ ARDS
**Does the patient meet ICU Admission Criteria?** *(Criteria linked below)*	Yes for **12 days**
**Current indications for admission to the ICU:**	Requires ventilatory support
**COVID Test Status:**	Positive ______
**Current level of respiratory support:**	Ventilator ______
**What degree of ARDS does the patient have?**	Mild (PaO2/FiO2 =200-300 with PEEP or CPAP≥5)
**What is the patient's response to current treatment?**	Improving = Is clinically improving

© 2022 Butler CR et al. *JAMA Network Open.*

eFigure 2. Prognostic Category Descriptions as Viewed by the Triage Team

Considerations for Priority Level		
Priority Levels	Likelihood to Survive to Discharge **with** Resource	Re-evaluation of Treatment
RED<br>Priority 1<br>Scarce Resource (SR) when available	≥90%	Improving
ORANGE<br>Priority 2<br>SR when available *after RED*	50-89%	
YELLOW<br>Priority 3<br>SR when available *after RED & ORANGE*	11-49%	
BLUE<br>Priority 4<br>SR when available *after RED, ORANGE, & YELLOW*	≤10%	Worsening
**Striped**<br>**Priority 5**<br>**SR when available *after RED, ORANGE, YELLOW, and BLUE***	Persons who have been diagnosed with one of the following conditions:<br>a. Severe acute trauma with low survival rate<br>b. Severe burns with low chance of survival*<br>c. Persistent vegetative state or coma	

*Saffle JR, Gibran N, Jordan M: Defining the ratio of outcomes to resources for triage of burn patients in mass casualties. *J Burn Care Rehabil* 2005; 26:478–482

© 2022 Butler CR et al. *JAMA Network Open.*
